# Role of offset and gradient architectures of 3-D melt electrowritten scaffold on differentiation and mineralization of osteoblasts

**DOI:** 10.1186/s40824-019-0180-z

**Published:** 2020-01-03

**Authors:** Naghmeh Abbasi, Saso Ivanovski, Karan Gulati, Robert M. Love, Stephen Hamlet

**Affiliations:** 1 School of Dentistry and Oral Health, Griffith University, Gold Coast Campus, Southport, Queensland 4215 Australia; 2 Menzies Health Institute Queensland, Griffith University, Gold Coast Campus, Southport, Queensland 4215 Australia; 3 School of Dentistry, University of Queensland, Herston Campus, St Lucia, Queensland 4072 Australia

**Keywords:** Melt electrowriting (MEW), Pore size, Scaffold, Polycaprolactone (PCL), Bone differentiation

## Abstract

**Background:**

Cell-scaffold based therapies have the potential to offer an efficient osseous regenerative treatment and PCL has been commonly used as a scaffold, however its effectiveness is limited by poor cellular retention properties. This may be improved through a porous scaffold structure with efficient pore arrangement to increase cell entrapment. To facilitate this, melt electrowriting (MEW) has been developed as a technique able to fabricate cell-supporting scaffolds with precise micro pore sizes via predictable fibre deposition. The effect of the scaffold’s architecture on cellular gene expression however has not been fully elucidated.

**Methods:**

The design and fabrication of three different uniform pore structures (250, 500 and 750 μm), as well as two offset scaffolds with different layout of fibres (30 and 50%) and one complex scaffold with three gradient pore sizes of 250–500 - 750 μm, was performed by using MEW. Calcium phosphate modification was applied to enhance the PCL scaffold hydrophilicity and bone inductivity prior to seeding with osteoblasts which were then maintained in culture for up to 30 days. Over this time, osteoblast cell morphology, matrix mineralisation, osteogenic gene expression and collagen production were assessed.

**Results:**

The in vitro findings revealed that the gradient scaffold significantly increased alkaline phosphatase activity in the attached osteoblasts while matrix mineralization was higher in the 50% offset scaffolds. The expression of osteocalcin and osteopontin genes were also upregulated compared to other osteogenic genes following 30 days culture, particularly in offset and gradient scaffold structures. Immunostaining showed significant expression of osteocalcin in offset and gradient scaffold structures.

**Conclusions:**

This study demonstrated that the heterogenous pore sizes in gradient and fibre offset PCL scaffolds prepared using MEW significantly improved the osteogenic potential of osteoblasts and hence may provide superior outcomes in bone regeneration applications.

## Introduction

Bone lesions that results from fracture, infections and tumors require targeted strategies for effective clinical treatment [1]. The combination of a three-dimensional scaffold combined with growth factors and/or cells has significant potential in bone tissue engineering as an ideal bone substitute option. However such scaffolds are not widely applied in clinical medicine, with the inability to seed a large enough cell population into scaffolds being one key impediment [2]. A desirable porous interconnected scaffold is required to allow uniform cell diffusion and distribution throughout the entire scaffold structure [3]. Many studies have attempted to solve this problem with various approaches, such as using bioreactors for enhanced cell proliferation, chemical and biological surface modification and varying the pore size of the scaffolds in a coordinated fashion to geometrically represent a cross section of a bone defect for an individual patient as a result of trauma or cancer metastasis [4–7]. Notably, previous studies showed that cell differentiation is influenced by the porosity, elasticity and stability of the scaffold [8].

Melt electrowriting scaffold fabrication enables precise control of the porosity, pore morphology and pore size of the printed scaffold architecture [9]. This allows predictable strain and stress distribution within the scaffold, which can be further optimized to produce an environment favouring higher cell infiltration through appropriate interconnectivity within the scaffold and subsequent vascularization and angiogenesis [10].

Previous studies have shown rapid bone formation was associated with pore sizes between 290 and 310 μm. While smaller pore sizes enhance the mechanical properties of the scaffold, the optimal size for vascularization was noted to be ~ 400 μm [11, 12]^.^ Therefore, fabrication of a ‘gradient’ porous scaffold, whereby the pore size gradually increases from one layer to the next, may overcome some of the individual limitations of both small and large homogeneous pore size scaffolds.

Gradient structures provide macropores suitable for vascularization, efficient gas/waste diffusion and nutrient supply at the expense of reduced mechanical stability. However, the denser structure of a heterogeneous gradient architecture improves ion signalling and protein adsorption as well as mechanical properties of the scaffolds, such as compressive strength. Additionally, they facilitate greater protein adsorption and better cell adhesion which decreases in the larger pore size of a homogeneous scaffold [13]. Interestingly, the gradient scaffold architecture is similar to the natural structure of native bone tissue that represents different mineral density from cancellous bone to cortical bone [14, 15].

Furthermore, higher pore size in gradient scaffolds has been shown to enhance permeability, cell migration, as well as sufficient nutrients and oxygen tension in larger pores and up-regulate osteopontin and collagen type I expression, thus generating more bone mass, vascularization and blood vessel ingrowth while inhibiting the formation of cartilaginous tissue in the regenerating sites [16, 17]. On the other hand, the smaller size of gradient pores promote cell seeding and growth by providing higher surface area [18].

The complex heterogeneous and hierarchical structure of bone tissue creates significant variation in the compressive and tensile strength of different regions within bone. It’s been shown that the morphology of the pores influences the mechanical properties and the structure of the scaffolds i.e. with more complex morphological architectures, the compressive strength will be increased [19], however, the scaffolds with greater Young’s modulus and smaller pore size are preferred in applications required to withstand greater loads [20]. According to Sobral et al., the simple architecture of homogeneous scaffolds is prone to collapse under high stress applied to the scaffolds, while the complexity of non-uniform porous scaffolds enables them to recover after deformation and maintain their elastic state which is critical for biomaterials implanted for bone applications [21].

Our previous studies have shown that, when compared to simpler homogeneous structures, scaffolds with offset and gradient architectures resulted in significantly higher cell proliferation following seeding (Additional file 1) [22]. As a logical extension of this work, this study will investigate the potential effects of heterogeneous porous scaffold architectures, particularly offset and gradient structures on osteogenic gene expression by osteoblasts seeded into these scaffolds and the rate of mineralization throughout the construct.

## Methods

### Preparation of microfibrous PCL membrane

**Fig. 1:**
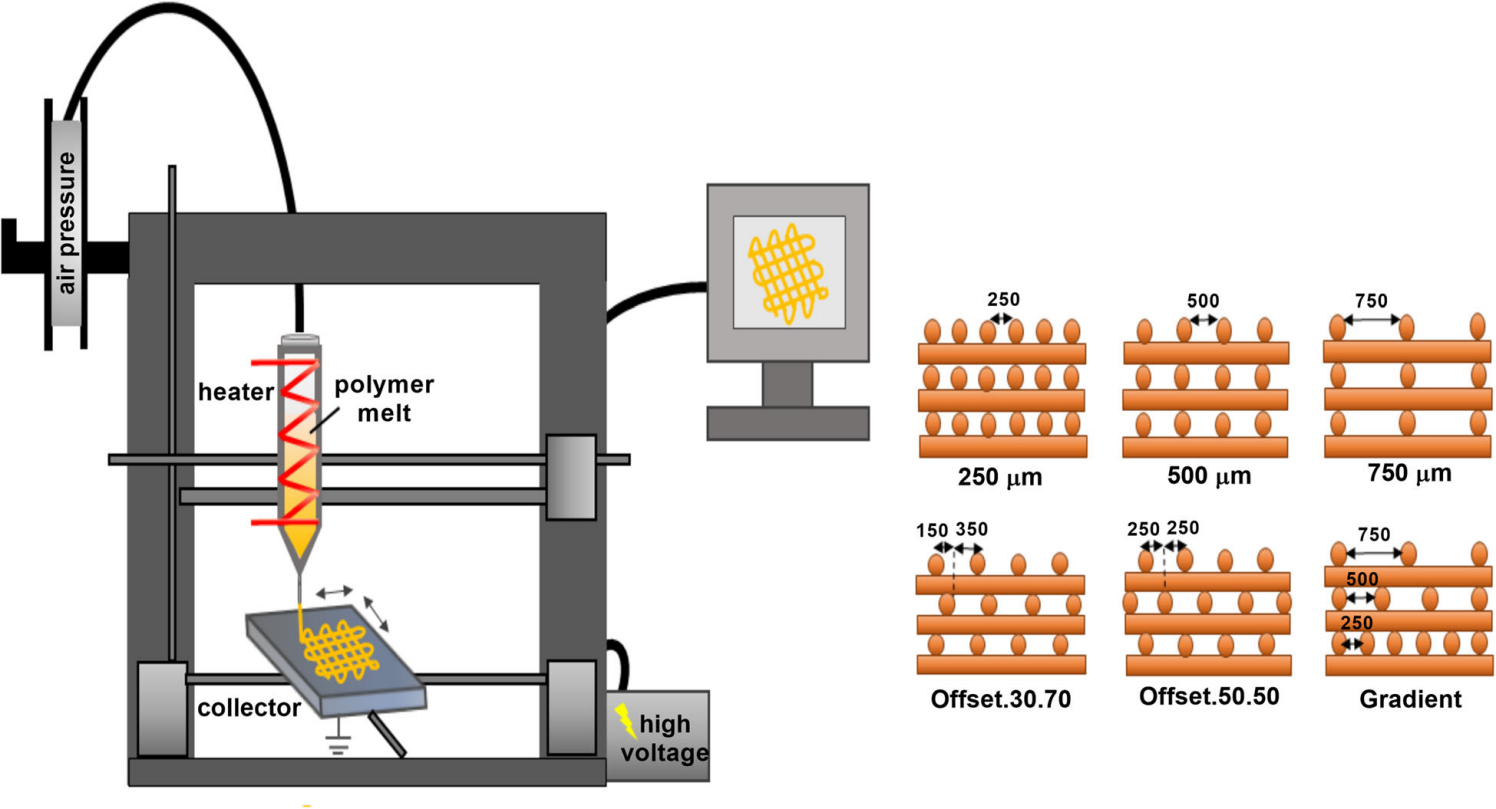
Schematic illustrating the preparation of the scaffolds with different architectures by using MEW method

A melt electrowriter (MEW) system was used to produce a fibrous scaffold with the fibre diameter in a range of 6–10 μm. The parameters for the components nozzle diameter, voltage, temperature and feeding rate were described in as previously reported [22]. Two different scaffold structures; homogeneous pore size (250 μm, 500 μm, 750 μm) and heterogeneous architecture including a tri-layer scaffolds with different pore sizes from 250 μm on top, 500 μm in the middle, and 750 μm at the bottom of the scaffold and offset scaffolds in which layers were printed with various offset values of 30 and 50% compared to the previous layer (Fig. 1).

Subsequent calcium phosphate (CaP) coating was perform using simulated body fluid (SBF). The surface modification was achieved in three steps; 1) pre-treatment of the samples in 1 M NaOH aqueous solution for surface activation for 0.5 h 2) immersion of the samples in 10X SBF at 37 °C for 1 h 3) soaking the samples in 0.5 M NaOH for 0.5 h to obtain an uniform coating then rinsing with distilled water and allowing to dry.

### Cell culture

Human osteoblast cells (hOB) obtaining from alveolar bone of a healthy female were seeded onto the scaffolds (2 × 10^4^ in 15 μl) and cultured in either osteogenic or basal medium for 3, 14 and 30 days, according to previously reported protocols [22].

### Scanning electron microscopy (SEM)

Cell morphology after 30 days culture on the electrowritten scaffolds was captured by scanning electron microscopy (SEM). The substrates from cell culture were fixed in 3% glutaraldehyde, then dehydrated by treating in 0.1 M Cacodylate buffer three times, each 10 min and post-fixed with 1% Osmium Tetroxide for 1 h. After that, the specimens were washed in Milli-Q water two times, each 10 min. The samples were then rinsed in a concentration gradient of ethanol (40–100%) 10 min for each. To visualise cell penetration into the scaffolds, cross-sections of the samples were fragmented in liquid nitrogen in the middle stages of the dehydration process. Finally, hexamethyldisilazane (HMDS, Sigma *Aldrich*, UK) was used to replace the critical point drying step in biological samples for two times, each 30 min. The samples were mounted on the Al stubs using adhesive carbon tapes and were Au-coated for observation by the SEM (Zeiss Sigma FESEM).

### Alkaline phosphatase (ALP) activity

The concentration of alkaline phosphatase (ALP) in cells was measured after 3, 14 and 30 days of culture using p-nitrophenyl phosphate as a phosphatase substrate in a commercial Alkaline Phosphatase colorimetric assay Kit (Sigma Chemical, St. Louis, MO, USA) at a wavelength 405 nm according to the manufacturer’s instructions. The ALP concentration was subsequently normalised to the total protein content (Quick Start Bradford protein assay, Bio-rad, Australia) of each sample.

### Alizarin red staining (ARS)

To assess the osteogenic potential of the osteoblasts cultured in the melt electrowritten scaffolds with different pore sizes, alizarin red S staining of calcium deposited in the extracellular matrix of osteoblasts cultured on the scaffolds after 14 and 30 days was carried out. Briefly, the culture media was removed and the scaffolds washed using PBS. The cells within the scaffolds were fixed in 4% paraformaldehyde then placed in 500 μL of Alizarin Red S solution (40 mM) for 20 min at room temperature. After washing, the stained cells and scaffolds were imaged using an inverted phase contrast tissue culture microscope (Olympus, CKX 41, NY, USA). Quantification of these results was achieved by dissolving the samples in 1 mL 10% Cetylpyridinium Chloride for 1 h before transferring to 96 well plates and measuring the absorbance at 540 nm in a microplate reader (POLARstar Omega, BMG LABTECH, Germany). The average value of the negative controls (scaffold without cells) [22] was subtracted from the values of the corresponding experimental groups.

### Micro-computed tomography (μ-CT) of cell-scaffold construct

To further evaluate mineralisation within the cell-seeded scaffolds, μ**-**CT analysis of constructs cultured in osteogenic differentiation medium was compared to the control group (basal medium) after 30 days of culture. The volume of mineralisation was measured by the μ-CT software (μCT40, SCANCO Medical AG, Brüttisellen, Switzerland). The average value of the negative controls (cell seeded scaffolds in expansion media) was subtracted from the values of the corresponding experimental groups. The sample was put inside the tube containing deionised water so that, it was located at the bottom of tube and cotton wool was used to stop moving of sample during scanning process. The tube was sealed to prevent solution evaporation. For scanning, the high-resolution mode was selected and the X-ray tube was applied at 45 kVp and 177 lA. Integration time was set to 300 m sec and a three-fold frame averaging was applied using the same μ-CT hardware, acquisition, and reconstruction parameters as above. Three-dimensional images of the scaffolds were reconstructed by the software package.

### Real-time PCR analysis (q-PCR)

RNA was extracted from the osteoblast cells 14 and 30 days after seeding onto the scaffolds using a TRIzol extraction kit (Ambion, USA). Following cDNA synthesis from 100 ng of total RNA (Taqman cDNA synthesis kit, Life Technologies, USA), the expression of osteopontin (*opn*), osteocalcin (*ocn*), bone morphogenetic protein 2 (*bmp-2*), alkaline phosphatase (*alp*), collagen type Ia (*col Ia*), wingless-related integration site (*wnt*) family member 3a (*wnt3a)*, and wnt family member 5a (*wnt5a)* was achieved with an ABI 7900HT real-time PCR system (Life Technologies, USA) using SYBR Green Real-Time PCR Master Mix (Life Technologies, USA) and the following protocol; 3 min at 95 °C for polymerase activation followed by 40 cycles of 10 s denaturation at 95 °C, 20 s annealing at 58 °C, and 1 s extension at 72 °C. *β-actin* expression was used as a house keeping gene and for normalization of the data.

### Immunofluorescence staining

For immunocytochemical analysis of the osteoblast cells cultured on different pore size PCL scaffolds (tissue culture plate acted as the control group) for 14 and 30 days, cells were fixed with 4% paraformaldehyde in PBS (Polysciences, Warrington, PA, USA) for 30 min at room temperature then gently rinsed with PBS. The cell membranes were then permeabilized and blocked with a protein blocker solution (1% BSA, 22.52 mg Glycine in 0.1% Tween 20 in PBS), Sigma Aldrich) for 30 min. After washing, the cells were incubated in the following diluted primary antibodies at 4 °C overnight: mouse monoclonal anti-Collagen IA (1:250, SantaCruz Biotechnology, USA), rabbit polyclonal anti-Collagen III (1:100, abcam, Australia), mouse monoclonal anti-Osteocalcin (1:200, abcam, Austrailia).

The cells were rinsed in PBS (three times, 5 min per wash) and incubated in the appropriate secondary antibody i.e. Alexa Fluor 488-conjugated goat anti-rabbit (1:200, abcam, Australia) or F (ab^`^)2-Goat anti-Mouse IgG FITC (1:200, ThermoFisher Scientific, USA) at room temperature in the dark for 1 h. Cell nuclei were stained using 40, 6-diamidino-2-phenylindole (DAPI, Vector Laboratories, Burlingame, CA, USA) in PBS (1:1000) for 30 min. The samples were mounted onto glass slides for visualisation using a fluorescence microscope (Nikon, Eclipse- Ti, U.S.A).

### Statistical analysis

Statistical analysis of any differences between means was performed using a two-way ANOVA with correction for multiple comparisons. The experiments were run in triplicate. A *p*-value of < 0.05 was considered as statistically significant.

## Results

### Cell-scaffold morphology

**Fig. 2:**
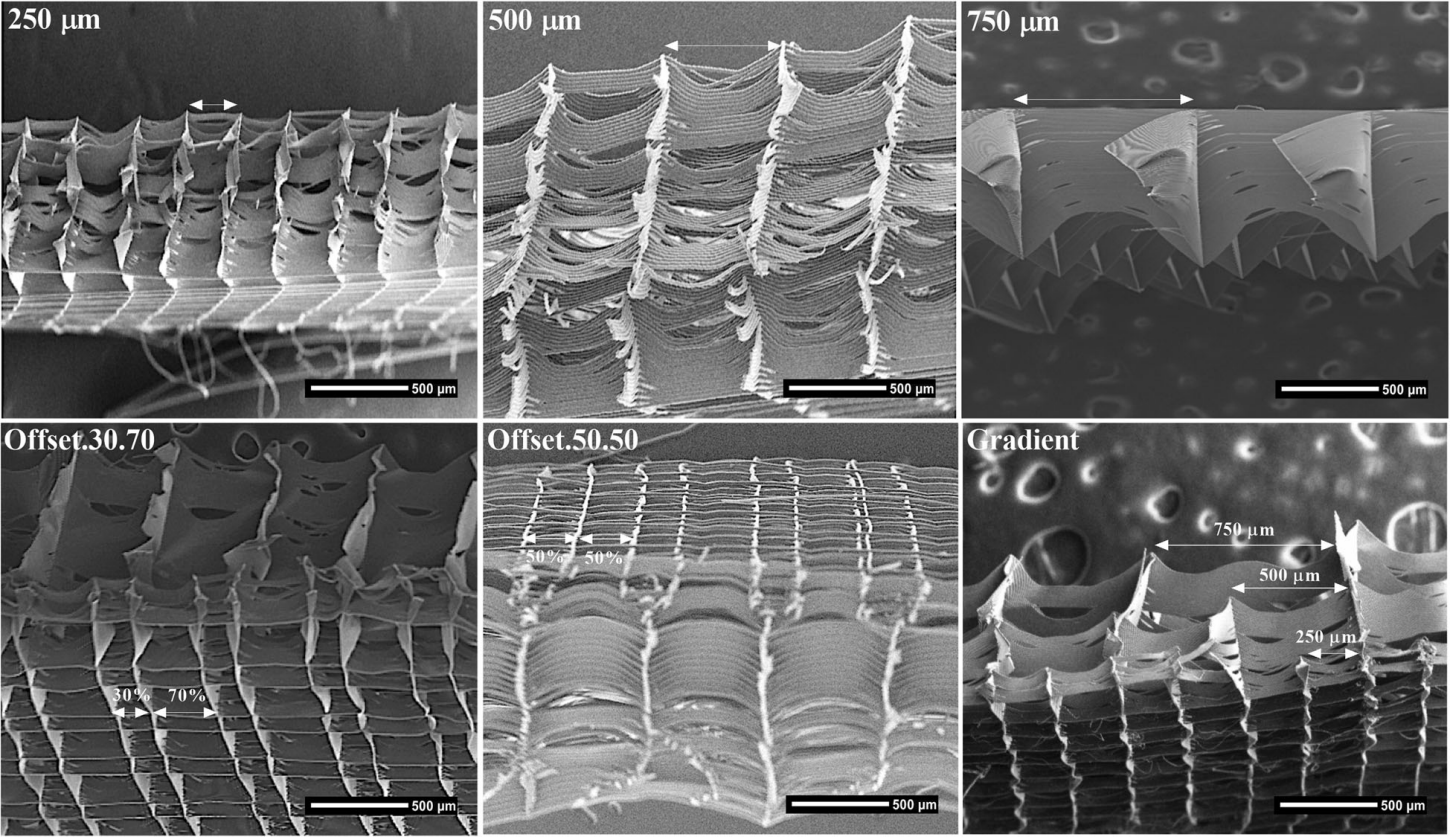
SEM image of the porous scaffold structures

**Fig. 3:**
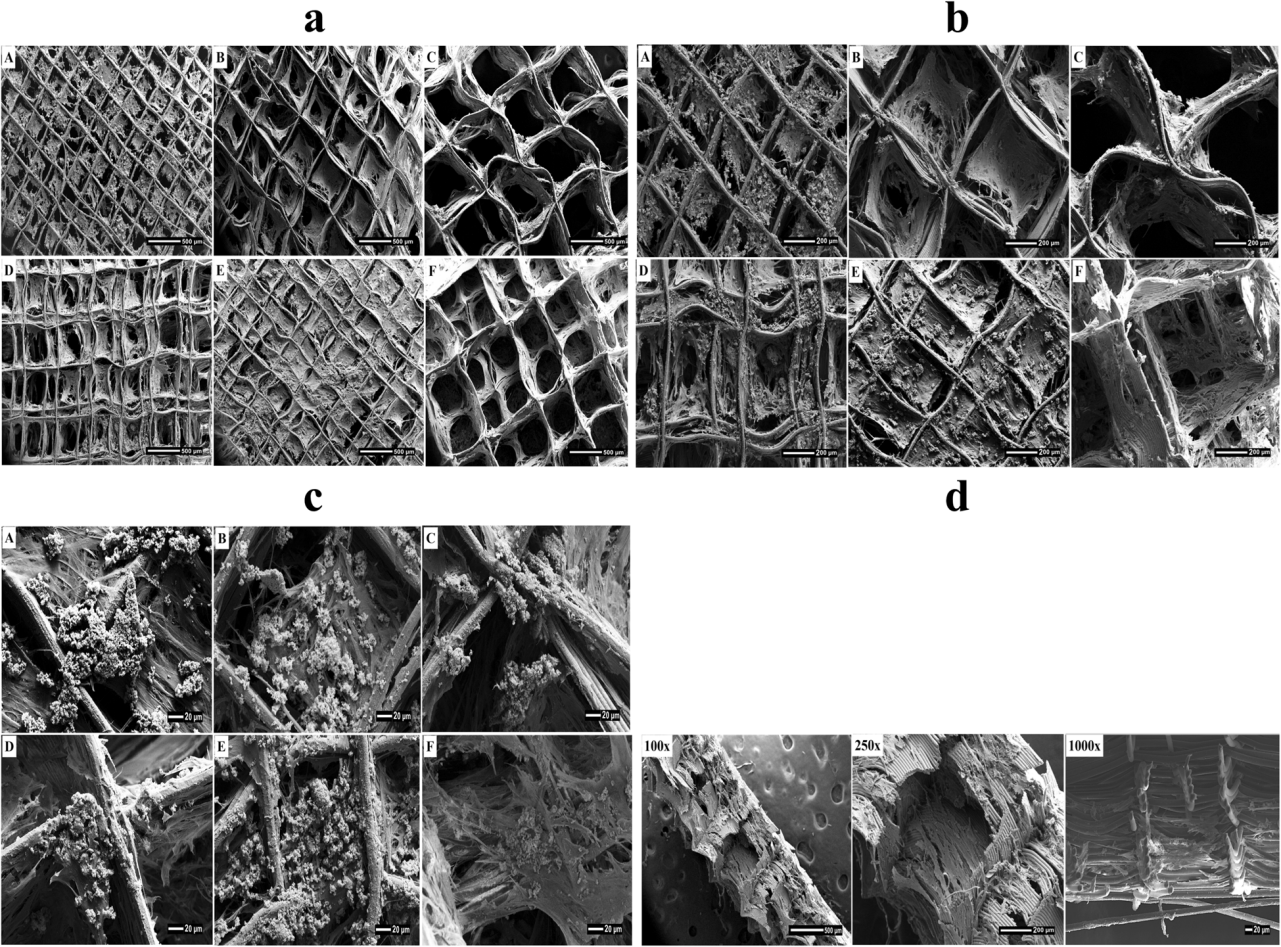
SEM micrographs showing different magnification (**a**) 100X, (**b**) 250X and (**c**) 1000X of the morphology and mineralization of hOB on the electrowritten PCL scaffolds after 30 days of culture a: 250 μm; b: 500 μm; c: 750 μm; e: Offset.30.70; e: Offset.50.50; f: Gradient. **d** Cross-sectional view of gradient scaffold structure

Figure 2 SEM images show the morphology and pore arrangement of the MEW PCL scaffolds prior to cell seeding. Thirty days following seeding with osteoblasts, SEM images showed good attachment and growth of the osteoblasts onto the PCL scaffolds (Fig. 3a, b, c). Optimum cell attachment and proliferation was identified on the 250 μm pore size homogeneous scaffold and the offset architecture scaffolds where the majority of cells appeared to be entrapped in the space between two displaced fibres in the offset groups. The 500 μm and 750 μm pore sizes appeared to be too large for the cells to interact with the scaffold fibers whereas in the gradient group the cells passed through the largest pore size at the top of scaffold then settled down into the smaller pores at the bottom of the construct (Fig. 3c)

Under lower magnification, information on the degree of scaffold mineralization and the position of the cells within this mineralized matrix after 30 days culture can be observed (Fig. 3a, b). Reflecting the cell attachment results, calcified matrix production occurred predominately on the offset and 250 μm surface scaffolds. At higher magnification, globular shaped deposits could be distinguished in the vicinity of the more dense regions of cell-fibre junctions and in the smaller areas between the pores (Fig. 3c). In the gradient group, the cells appeared more concentrated at the bottom of scaffold structure having passed through the large pore size at the top of scaffold and settled down into the smaller pores at the bottom of scaffold (Fig. 3d).

### Impact of porous scaffolds on ALP activity of osteoblast cells

**Fig. 4:**
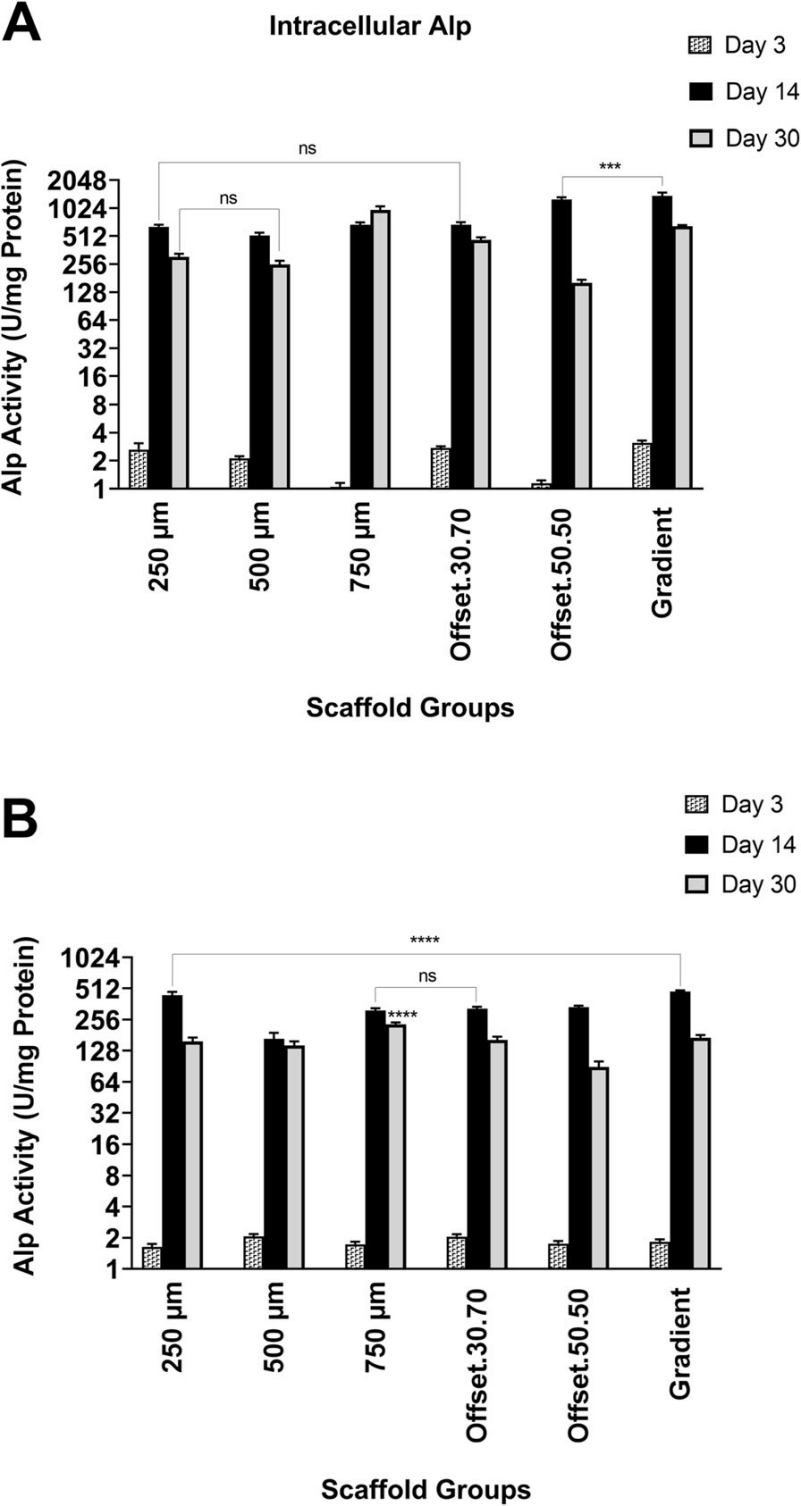
ALP activity of osteoblasts in osteogenic (**a**) and basal (**b**) medium seeded on PCL scaffolds with various porous structures for 3, 14, and 30 days; ns: non-significant; *: significant versus other scaffolds at the same time point (*p* < 0.01)

Alkaline phosphatase (ALP) activity was very low at day 3 in all groups (Fig. 4a, b) in the similar ranges between 0.7 to 3.1 that the maximum amount was displayed for gradient, 250 μm and offset.30.70 scaffolds respectively. By day 14 ALP activity had increased in all groups with the highest levels of activity in osteoblasts cultured on the offset.50.50 and gradient scaffolds. However, it was peaked significantly for the gradient structure culturing in osteogenic media compare to the other groups. By 30 days of culture, a reduction of ALP activity was seen for all groups except the homogeneous 750 μm pore size scaffolds.

### Influence of porous scaffolds on calcium deposition by osteoblast cells

**Fig. 5:**
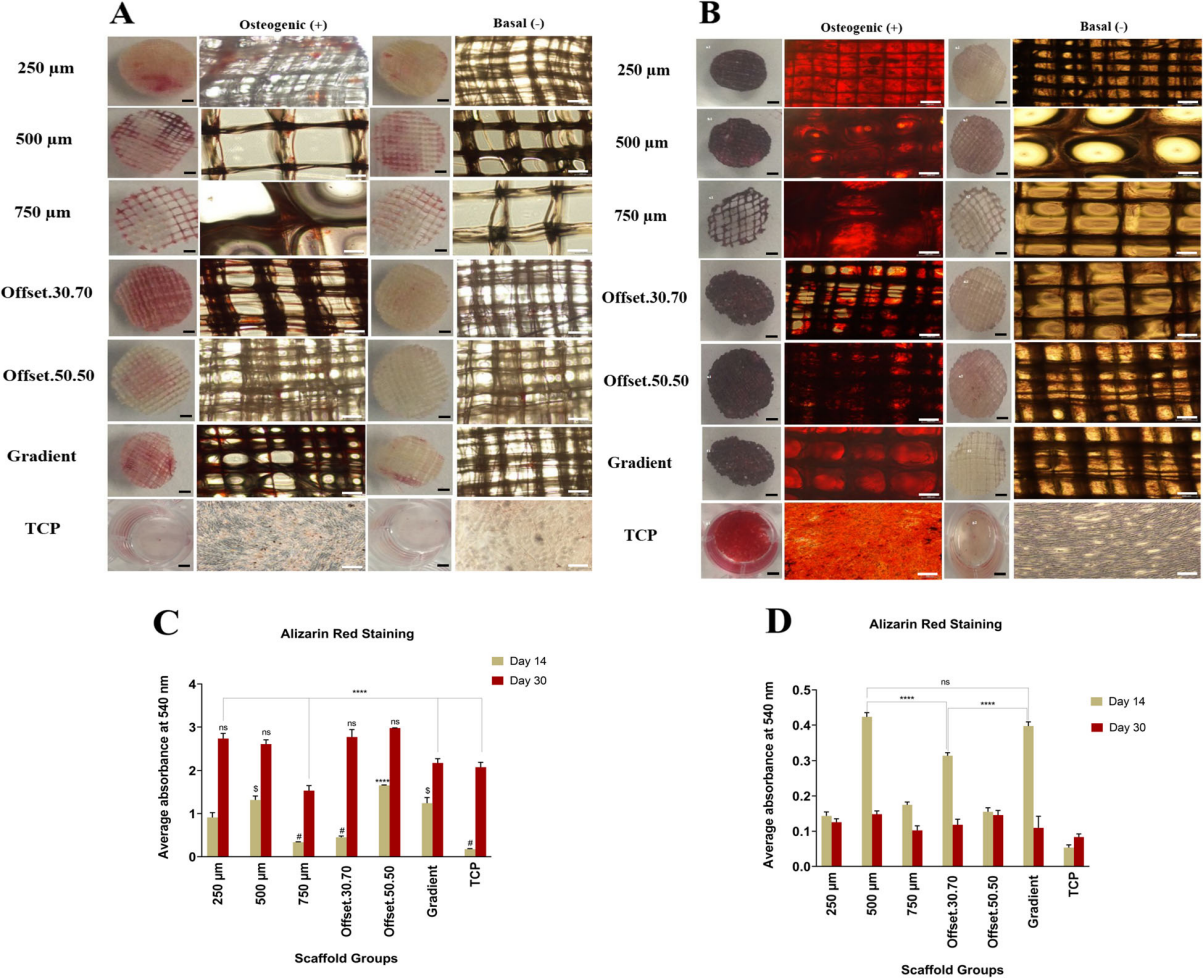
Alizarin Red staining of osteoblast cells seeded on porous PCL scaffolds maintained for (**a**) 14 and (**b**) 30 days in basal and osteogenic medium; Quantitative analysis of Alizarin Red staining in osteogenic (**c**) and basal (**d**) medium by measuring the optical density (OD); ns, # and $: non-significant; *: significant versus other scaffolds having the same sign (*p* < 0.01); (Black scale bar = 1 mm, White scale bar = 100 μm)

To assess mineralized matrix formation on the scaffold, analysis of calcium deposition was performed after 14 and 30 days of culture in osteogenic and basal medium (Fig. 5a, b). The analysis of alizarin red staining showed a significant difference in calcium accumulation in the scaffolds cultured in osteogenic differentiation medium compared to basal medium as the control group. The data indicated that all scaffolds were able to significantly induce osteogenic differentiation through the augmentation of calcium in the extracellular matrix. The calcium deposition started to appear after 14 days and gradually increased up to 30 days in osteogenic medium. Figures 5c, d showed the quantitative analysis of mineralization in all of the groups where the average calcium deposition was higher on offset.50.50, offset.30.70 and 250 μm PCL scaffolds respectively. The 750 μm showed the lowest amount after 30 days.

### Impact of porous MEW scaffolds on osteoblast cells mineralization

**Fig. 6:**
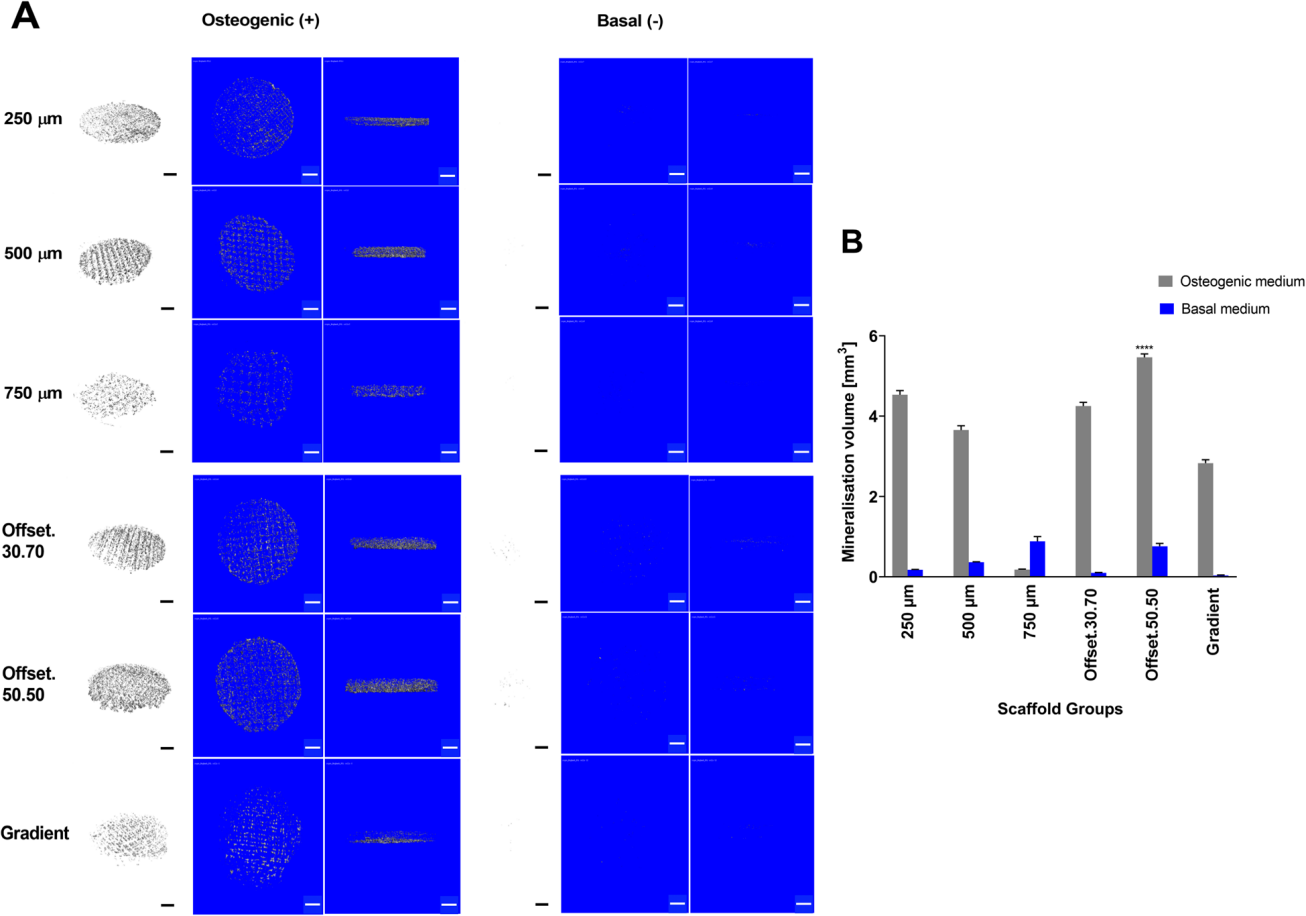
ECM mineralization indicated in grey and the micro-CT images were segmented from each other in 2D and 3D images. **a** Micro-CT analysis of the 3D cell/scaffolds mineral deposition after 30 days cultured in osteogenic and basal medium. **b** Quantitative comparison of the mineral volumes/total volume ratio within the scaffolds. *: significant compared to other groups (*p* < 0.0001); (Scale bar = 1 mm)

Figure 6 compares extracellular matrix mineralisation of osteoblasts seeded onto the scaffolds and cultured in osteogenic media with those cultured in basal media. As expected from the corresponding alizarin red staining data, mineralization was distributed within the pores of the scaffold (Fig. 6a). Consistent with the alizarin red staining, pixel quantification of the μ-CT images (Fig. 6b) also confirmed that the offset.50.50 scaffold had the highest amount of mineralisation while the 750 μm pore size scaffold showed the lowest level of mineralization.

### Effect of porous scaffolds on osteoblast gene expression of osteogenic markers

**Fig. 7:**
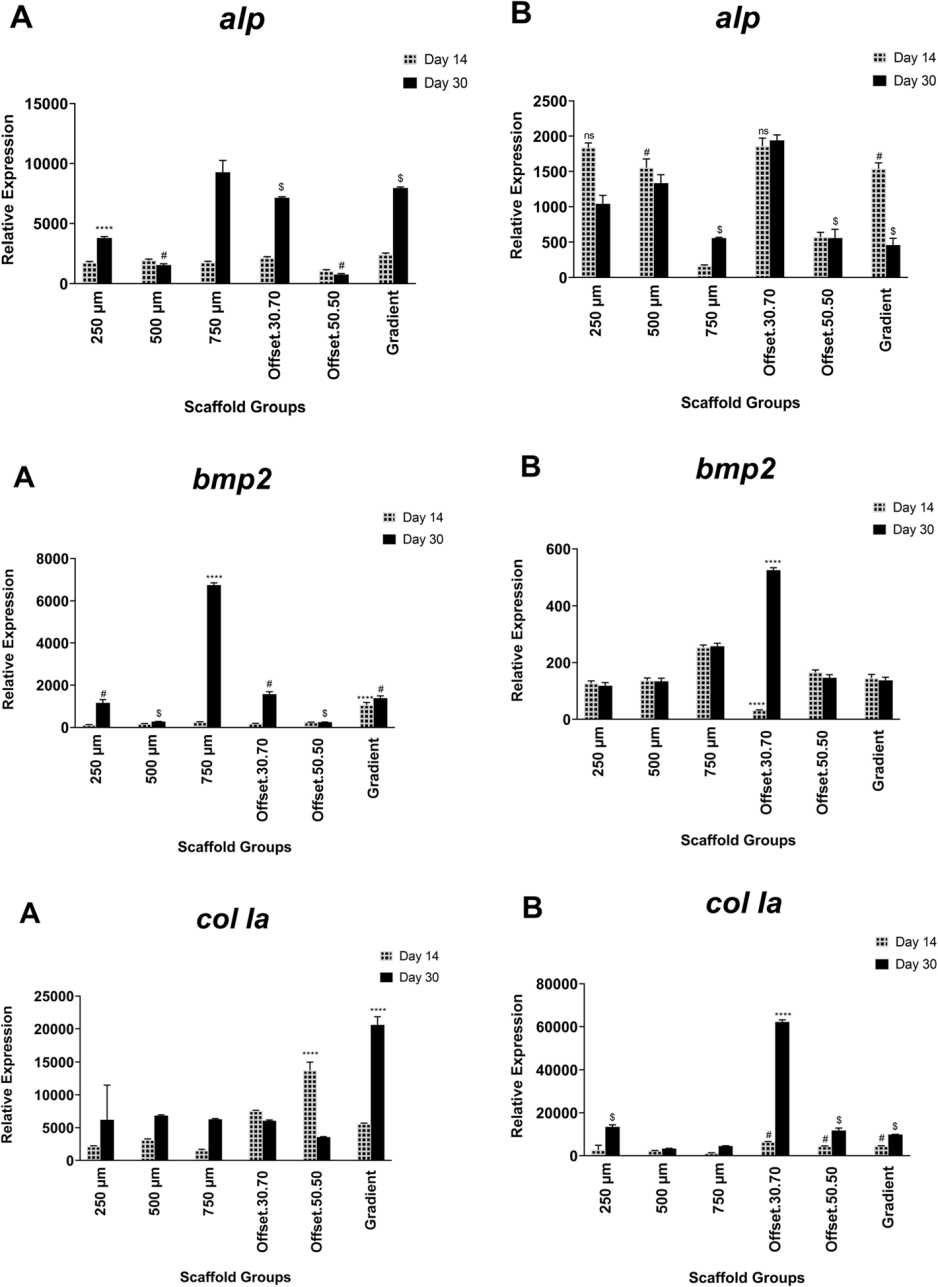
Gene expression pattern during mineralization of human osteoblast cells seeded on PCL scaffold structures in osteogenic (**a**) and basal (**b**) medium for 3, 14, and 30 days Expression of genes was analyzed by real- time PCR and normalized to the levels of *β-actin*. ns, # and $: non-significant; *: significant versus other scaffolds having the same sign (*p* < 0.0001)

**Fig. 8:**
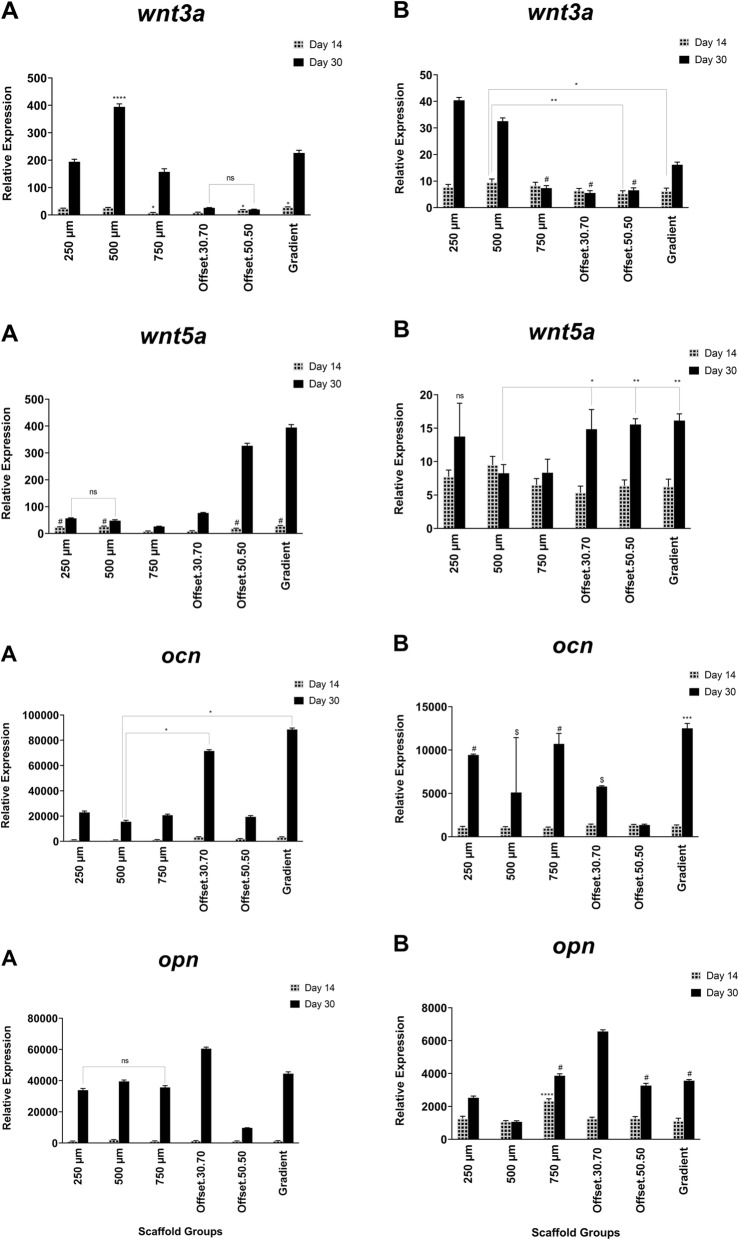
Gene expression pattern during mineralization of human osteoblast cells seeded on PCL scaffold structures in osteogenic (**a**) and basal (**b**) medium for 3, 14, and 30 days Expression of genes was analyzed by real- time PCR and normalized to the levels of *β-actin*. ns, # and $: non-significant; *: significant versus other scaffolds having the same sign (*p* < 0.0001)

Expression of the osteogenic markers *col Ia*, *alp*, *ocn* and *opn,* as well as the osteogenesis associated signalling molecules *bmp-2*, *wnt3a* and *wnt5a,* in osteoblasts cultured on the scaffolds were analysed by quantitative real-time PCR (Figs. 7, 8). After 14 days of culture in osteogenic medium, *col Ia* transcript levels were significantly higher compared to the other genes. Also, the expression of *col Ia* increased in offset.30.70 and 50.50 scaffolds on day 14. Among the different pore sizes, the gradient architecture induced the highest expression level of *wnt5a* and *ocn*. The expression of *alp* was not significantly different between the groups, except for 750 μm which showed the maximum level, while the lowest expression was observed for offset.50.50. The *alp* expression was up-regulated in offset.30.70 and gradient scaffold groups, but this was not statistically significant compared to the other groups. Following 30 days of cell culture, the mineralization-related markers *ocn* and *opn* were up-regulated. In all groups, the expression of *alp* was less than *ocn* and *opn* after 30 days. Therefore, all of the scaffold groups stimulated the upregulation of *alp* and *col I* expression at early stages of osteogenic differentiation after 14 days, while, the gradient and offset.30.70 scaffolds were able to express *ocn* and *opn*. Increase of *bmp2* gene expression was observed in 250 μm, offset.30.70 and gradient scaffolds, and at the highest quantity in the 750 μm group, compared to other groups. The assessed data demonstrated the high expression of *wnt5* in the gradient and 50.50 offset scaffolds.

### Osteoblast-related protein expression

**Fig. 9:**
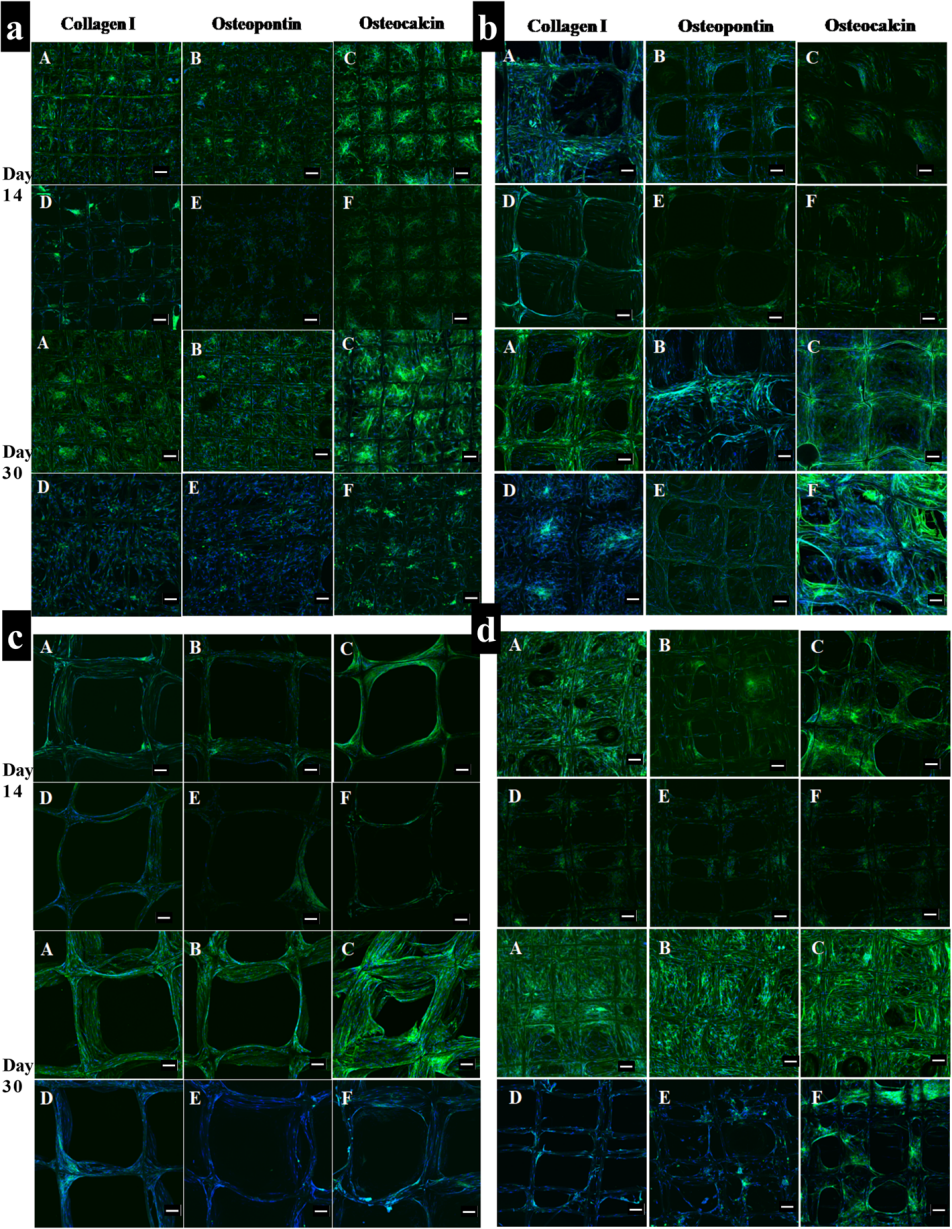
Immunocytochemistry analysis of osteogenitor markers (collagen I, osteopontin and osteocalcin) for human osteoblast cells cultured in: (a, b, c, d) Osteogenic medium (d, e, f) Basal medium after 14 and 30 days; **a**: 250 μm; **b**: 500 μm; **c**: 750 μm; **d**: Offset.30.70. Merge: Hoechst staining of the nucleus (blue), staining of the antibodies (green fluorescence); (Scale bar = 100 μm)

**Fig. 10:**
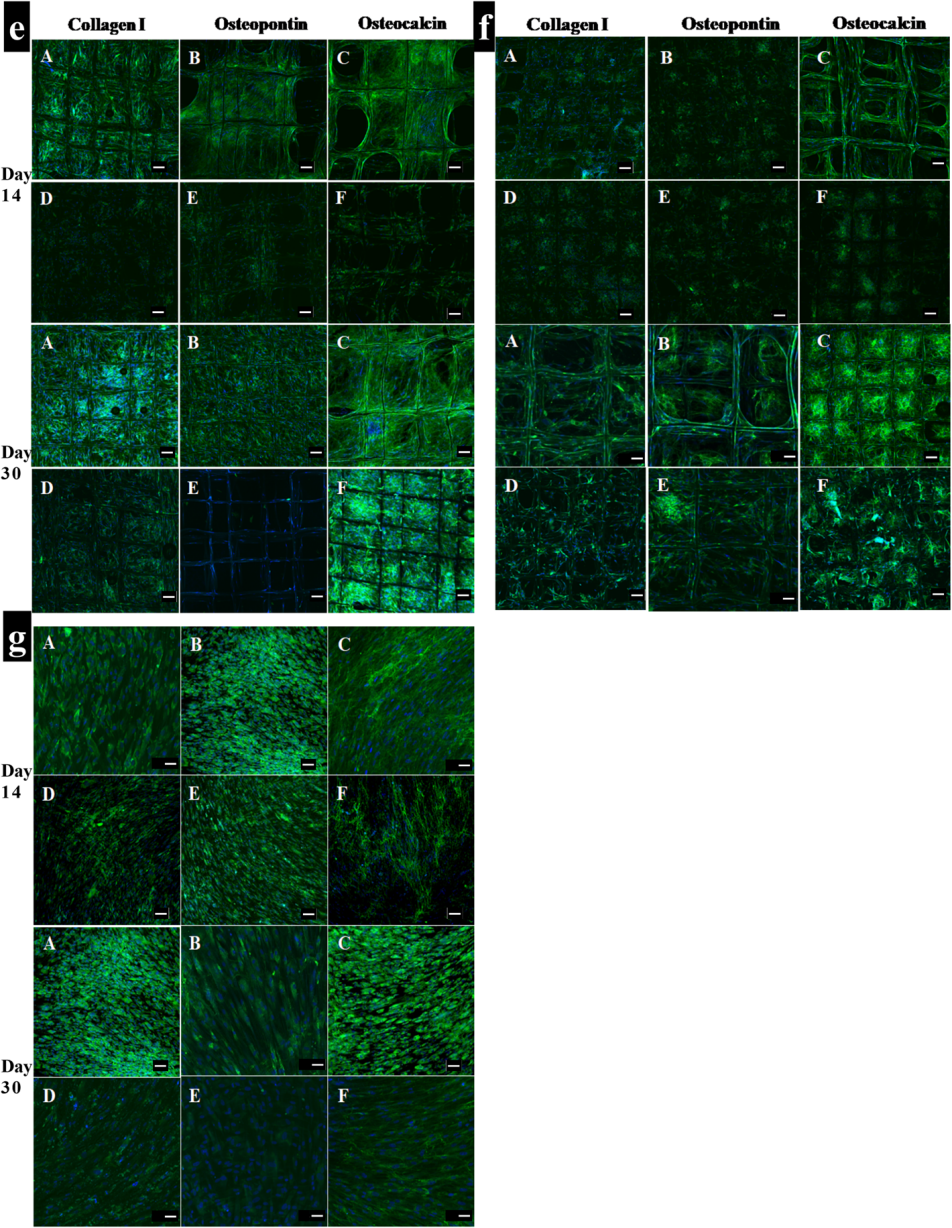
Immunocytochemistry analysis of osteogenitor markers (collagen I, osteopontin and osteocalcin) for human osteoblast cells cultured in: (a, b, c) Osteogenic medium (d, e, f) Basal medium after 14 and 30 days; **e**: Offset.50.50; **f**: Gradient; **g**: Tissue culture plate (TCP). Merge: Hoechst staining of the nucleus (blue), staining of the antibodies (green fluorescence); (Scale bar = 100 μm)

To evaluate the osteoconductivity of microporous melt electrospun PCL scaffolds, osteoblast cells were seeded on these scaffolds and cultured in osteogenic medium for 30 days in vitro. Immunocytochemistry was subsequently used to visualise collagen type I, collagen type III and osteocalcin deposition within the cellular matrix (Figs. 9, 10). Confocal microscopy demonstrated similar biocompatibility of the scaffolds as shown by the uniform spatial distribution of the cells on all six scaffold types. As expected, the images confirmed that collagen type I, collagen type III and osteocalcin were all expressed on all the scaffolds cultured in osteogenic media for 14 days and 30 days (Figs. 9, 10). These data indicated that all of the scaffolds showed markedly better expression of osteocalcin compare to the other two proteins. It was obvious that the number of differentiated cells on the scaffolds increased with time. The pore spaces were filled and covered completely by the cells after 30 days, particularly in gradient and offset scaffolds. However, the expressions of collagen type I, collagen type III and osteocalcin on the scaffolds cultured in osteogenic media were much higher than those on the scaffolds cultured in basal medium (Figs. 9, 10).

## Discussion

Several strategies can be utilized to attain porous scaffolds. As melt-electrowriting fabrication delivers a stable electrostatically drawn jet without whipping or random fibre deposition (as in solution electrospinning) it can be used for the formation of gradient structures mimicking native bone by precisely controlling fiber placement [23]. The proliferation and differentiation of cells within 3D scaffolds are affected by both the size and geometry of the scaffold’s pores [24] but it’s not clear however whether scaffolds with a uniform pore distribution of homogenous size, or constructs with a varying pore size distribution, are more suitable for bone regeneration applications. Therefore, the present study evaluated the effect of complex offset and graded porous structures on bone differentiation in contrast to the simpler homogeneous MEW PCL scaffolds.

According to the SEM observations, the presence of more anchorage points in offset and 250 μm structures allowed more cells to populate and grow compared to the other scaffolds, suggesting that the smaller pore volume induced more cell aggregation. Porous scaffolds with an offset designs creates a higher surface area that could result in more cell distribution and proliferation. Since the fibres positioning affected the flow path of the media during cell seeding, the cell attachment efficiency increased. This is due to the presence of the ‘obstacles’ in the heterogeneous (offset/gradient) pore design, which provides cells with additional anchorage points during the seeding process [25]**.** The enhanced cell growth in the smaller pore size scaffolds may also be attributed to the higher concave surfaces and curvature of the small pore size than the large pore size which results in minimizing the surface area and surface tension [26, 27].

Surface tension or surface energy comes from the tendency of cells to approach each other to achieve a balanced energy i.e. neighboring cells have the least energy and the most stable state. This is the natural tendency of molecules to minimize their energy [28], therefore, to reduce their surface energy, the cells will select the corners of the pore space to enhance contact with the other cells. Because of the lower angle between the fibers of small pore sizes, it can provide a more suitable environment for the cells to interact with each other, thus minimizing their residual energy and becoming more stable [29, 30].. This could be the reason for higher cell proliferation and faster growth in the smaller pores of 250 μm and offset.50.50 scaffolds with the higher curvature, compared to larger pore sizes of 750 μm.

While there are no experimental findings to support the hypothesis that minimal surface areas advance bone regeneration, concave surfaces compared to convex and planar surfaces have been shown to promote bone tissue regeneration, a process that increases with higher curvature [20, 31, 32]. This agrees with our study, which showed more mineralization in offset.50.50 and 250 μm scaffolds compared to the higher pore size of scaffolds.

Cells at the centre of the pores have the highest energy levels, and larger pore sizes generate more perimeter and thus less curvature [33]. This may be the reason for low cell density with larger pore sizes despite more penetration of the cells into the 500 μm and 750 μm pore size scaffolds after 24 h, compared to the 250 μm pore size scaffold. The restriction on infiltration though promotes differentiation over proliferation since the inner space is filled faster due to more interactions between cells in the smaller area, while larger pore sizes have more empty spaces promoting proliferation instead.

Our results are in agreement with the study of Di Luca et al., that also showed an increase in cell number and mineralization by reducing the pore size to 500 μm as well as in the area with the smallest pores (500 μm) of a complex four zone gradient (500/700/900/1100 μm zones) using PEOT/PBT (poly (ethylene oxide therephtalate)/poly (butylene therephtalate) and PCL scaffolds fabricated by rapid prototyping [34]. They also showed that the variety in cell density in the different regions of gradient scaffolds could be attributed to the increase of hypoxia inducible factor (HIF) in the higher hypoxic regions of small pore size where the oxygen levels could drop [35]. Previous studies already suggested that rising hypoxia leads to osteochondrogenic differentiation, mediated via HIF-1 and triggered through the elevation of ALP and OCN levels and mineralization [36–39]. Interestingly, we also found more calcium mineralization in smaller pore sizes of 250 μm and offset.50.50 structures.

Other models have shown that the behavior of cells can be affected by pore size. For example, chondrocytes produce large amounts of glycosaminoglycan (GAG) and collagen II in 400 μm sized pores, while they proliferate in 200 μm sized pores [40]. On the other hand, human mesenchymal stromal cells (hMSCs) seeded within PEOT/PBT scaffolds had significantly higher amounts of GAG in 500 μm pore size (non-gradient) and gradient (500–1100 μm) scaffolds, compared to largest pore sizes [41].

In the present study alkaline phosphatase activity, an early indicator of matrix mineralization, increased significantly after 14 days in the gradient and offset.50.50 scaffolds. After 30 days ALP activity decreased (compared to day 14) in all scaffolds, except for the 750 μm pore size scaffold. This suggests that the structures with the greater porosity of 750 μm and gradient architecture stimulate ALP expression. This finding is also in accordance with the in vitro study by Di Luca et.al (2016) which demonstrated that an increase in pore size of poly (ethylene oxide therephtalate)/poly (butylene therephtalate) (PEOT/PBT) and poly(ε-caprolactone) (PCL) scaffolds enhances ALP levels during human mesenchymal stromal cell differentiation [34].

Our study also showed that ALP levels were greater in 750 μm scaffold after 30 days compared to the other scaffolds. This finding was in accordance with the study of Hutmacher et.al (2000) that indicated the structures with greater porosity could stimulate ALP expression, since countering hypoxic conditions in the smaller pore size and unavailability of O_2_ and nutrient supply for the cells [42]. Kasten et.al (2008) demonstrated that the higher porosity of 65 and 75% β-tricalcium phosphate (TCP) ceramic scaffolds also enhanced ALP activity when compared to lower porosity (25%) scaffolds due to better nutrient and O_2_ transportation [43]. However, it also needs to be acknowledged that the higher levels of ALP activity after 30 days in the present study might also be the consequence of less cells initially attaching to the larger pore structure of the 750 μm scaffold.

Although higher porosity increased ALP activity, our results showed the offset scaffolds demonstrated superior matrix mineralization. This may be related to the surface wettability which increases surface free energy resulting in better cell attachment due to greater protein adsorption of ECM-products [44], and the rougher surface of the offset structures after calcium phosphate (CaP) coating modification, which was shown in our previous study by alizarin-red staining and surface area (BET) analysis [22]. It has been displayed the CaP coating on the surface of the fibres within the cells, that the higher level of mineralization was identified on the offset and then 250 μm scaffold structures through alizarin-red staining and micro-CT analysis. This is in agreement with the study of Hammerl et al. (2019) that showed that CaP/PCL scaffolds formed a mineralized matrix regardless of the cell type cultured on these scaffolds [45], suggesting that released ions and wide contact area between the cells and fluids can evoke higher mineralization in short term cell culture, although evidence for this phenomenon is not yet provided. Although some studies have used the commercial materials to create a biologically active surface of the implant [46, 47]. For example, Tontowi et al. reported the combination of Hydroxyapatite powder (HA-200, brand name of CAMCERAM II HA) and the mixture of Gelatin/ Polyvinyl Alcohol (PVA) [HA/G/PVA] fabricated by freeze-drying that resulted in the faster bone formation following 21 day of implantation [48].

The study of Yeo et al. (2012) also confirmed increased mineralization in 50 and 100% offset polycaprolactone (PCL) and β-tricalcium phosphate (β-TCP) scaffolds compared to no-offset structures [25]. Furthermore, higher hydrophilicity creates more surface free energy that results in better cell attachment due to the greater protein adsorption as more ECM-products are achieved by higher cell number [44]. Our results indicated a high level of *col Ia* gene expression in offset scaffolds over 14 days, which were expressed at the middle stage of differentiation. *Type I Collagen* gene expression, an early marker of osteoblast differentiation which results in increased bone mass in combination with OCN and ß-catenin functions [49], as well as increased up-regulation of *ocn* gene expression was indeed seen in offset.30.70 scaffolds after 30 days. Increased *bmp-2* gene expression was also observed in 250 μm, offset.30.70 and gradient scaffolds with the highest expression in 750 μm scaffolds. Activation of *bmp* signalling leads to osteocalcin and alkaline phosphatase expression [50] and after 30 days culture the activity of ALP increased in the 750 μm scaffolds compared to the other groups, while *bmp* expression was not upregulated.

The gradient scaffolds also had a high level of *wnt5* gene expression. The *wnt* family of secreted glycoproteins plays a critical role in bone formation, mediated through the expression of osteoblast- specific genes. Activation of this pathway also results in the expression of alkaline phosphatase, an early osteoblast marker associated with the Wnt pathway [51]. The gradient scaffold was associated with the highest expression levels of *wnt5* which correlated with increased *alp* expression in gradient scaffolds after 30 days of culture.

A previous study showed that up-regulation of bmp-2 supressed *wnt3a* signalling in MSCs and induced osteoblast differentiation [52]. Similarly, our results also showed higher expression of wnt3a in homogeneous 500 μm scaffold compared to bmp2, which may mean that the cells in this scaffold were stimulated towards proliferation instead of differentiation, in contrast to gradient and offset scaffolds which had lower wnt3a expression.

High expression of *ocn* and *opn* is associated with the late stages of differentiation and mineralization [53, 54]. Offset and gradient scaffolds had greater expression of *ocn* and *opn,* suggesting that these architectures enhanced osteoblast differentiation to exhibit the mature markers following 30 days. Sicchieri et al. (2012) also evaluated the effect of pore size on the osetogenic gene expression and showed that larger pore size of PLA-CaP scaffolds increased the expression of *alp*, *type I col* and *ocn*, similar to the higher expression of these markers in the gradient structure of this study, which may be influenced by the large pore size section in the gradient scaffold [55]. In the present study the increase in osteocalcin (OCN) expression especially in offset scaffolds suggests that this architecture could promote osteogenesis as osteocalcin modulates the matrix mineralization which is a later phase of osteogenic differentiation [56]. The findings of this in vitro study need to be confirmed in an appropriate in vivo model, to fully elucidate the effect of heterogeneous and homogeneous pore size of melt electrospun (MEW) scaffolds on bone regeneration.

## Conclusion

This study has shown that heterogenous offset and gradient porous scaffolds are favourable structures for bone differentiation compared with uniform pore size scaffolds. The gradient architecture with three different pore sizes (250–500-750 μm) favoured ALP activity, while scaffolds with an offset 50% value allowed more matrix mineralization and the expression of late osteogenic markers, such as osteocalcin, which promoted maturation of differentiated osteoblasts. Also, both the gradient and offset scaffolds appeared to support ECM deposition in contrast to the homogeneous porous scaffolds. Taken together, the finding of this research demonstrated the gradient pore size and the offset architecture of MEW scaffolds are able to overcome the limitation of small and large uniform pore sizes associating with cell adhesion and mineralization during the in vitro bone differentiation process.

## Supplementary information

**Additional file 1. **Osteoblast Proliferation. Proliferation of osteoblasts in osteogenic (+) and basal medium (−) seeded on PCL scaffolds with different porosity for 1, 3, 14, and 30 days. * significant versus other scaffolds. Δ nonsignificant versus 250 μm -. # and $ nonsignificant versus Gradient - (*p* < 0.01); (Reproduced with permission from Abbasi et.al. doi: https://doi.org/10.1021/acsbiomaterials.8b01456).

## Data Availability

The datasets used and/or analyzed supporting the conclusions of current study are available and will be presented by the corresponding author on reasonable request.

## Abbreviations

ALP: Alkaline phosphatase
ANOVA: Analysis of variance
ARS: Alizarin red staining
BET: Brunauer−Emmett−Teller
Bmp-2: Bone morphogenetic protein 2
CaP: Calcium phosphate
Col Ia: Collagen type Ia
DAPI: 6-diamidino-2-phenylindole
GAG: GlycosaminoglycanGlycosaminoglycan
HIF: Hypoxia inducible factor
HMDS: Hexamethyldisilazane
hMSCs: Human mesenchymal stromal cellsstromal cells
hOB: Human osteoblast cells
MEW: Melt electrowriting
Ocn: Osteocalcin
Opn: Osteopontin
PCL: Poly(ε-caprolactone)
PEOT/PBT: Poly (ethylene oxide therephtalate)/poly (butylene therephtalate
q-PCR: Real-time PCR analysis
SBF: Simulated body fluid
SEM: Scanning electron microscopy
TCP: Tissue culture plate
TCP: β-tricalcium phosphate
Wnt3a: Wingless-related integration site family member 3a
Wnt5a: Wingless-related integration site family member 3a
μ-CT: Micro-computed tomography
