# Supplementary material for: Role of offset and gradient architectures of 3-D melt electrowritten scaffold on differentiation and mineralization of osteoblasts
Source: Biomater Res. 2020 Jan 3;24:2. doi: 10.1186/s40824-019-0180-z (PMC6942301; doi:10.1186/s40824-019-0180-z)
Supplement: Supplementary file 1 — Additional file 1. Osteoblast Proliferation. Proliferation of osteoblasts in osteogenic (+) and basal medium (−) seeded on PCL scaffolds with different porosity for 1, 3, 14, and 30 days. * significant versus other scaffolds. Δ nonsignificant versus 250 μm -. # and $ nonsignificant versus Gradient - (p < 0.01); (Reproduced with permission from Abbasi et.al. doi: https://doi.org/10.1021/acsbiomaterials.8b01456). [file 40824_2019_180_MOESM1_ESM.zip › Additional file 1.docx]

**Role of Offset and Gradient Architectures of 3-D Melt electrowritten Scaffold on Differentiation and Mineralization of Osteoblasts**

**Naghmeh Abbasi ^1, 3*^, Saso Ivanovski ^2^, Karan Gulati ^2^, Robert M. Love^1^, Stephen Hamlet ^1, 3*^**

*^1^ School of Dentistry and Oral Health, Griffith University, Gold Coast Campus, Southport, Queensland 4215, Australia.*

*^2^ School of Dentistry, University of Queensland, Herston Campus, St Lucia, Queensland 4072, Australia*

*^3^ Menzies Health Institute Queensland, Griffith University, Gold Coast Campus, Southport, Queensland 4215, Australia.*

**Corresponding Authors:**

Stephen Hamlet

Griffith University, Gold Coast Campus, QLD, Australia, 4222

Ph: +61(7)56780483

E-mail: [s.hamlet@griffith.edu.au](mailto:s.hamlet@griffith.edu.au).

Naghmeh Abbasi

School of Dentistry and Oral Health, Griffith University, Gold Coast Campus, QLD, Australia, 4222

Ph: +61415937467

E-mail: [naghmeh.abbasi@griffithuni.edu.au](mailto:naghmeh.abbasi@griffithuni.edu.au) , [naghme.k@gmail.com](mailto:naghme.k@gmail.com)

**Additional file 1.** Proliferation of osteoblasts in osteogenic (+) and basal medium (−) seeded on PCL scaffolds with different porosity for 1, 3, 14, and 30 days. * significant versus other scaffolds. Δ nonsignificant versus 250 μm -. # and $ nonsignificant versus Gradient - (p < 0.01); (Reproduced with permission from Abbasi et.al. doi: 10.1021/acsbiomaterials.8b01456)


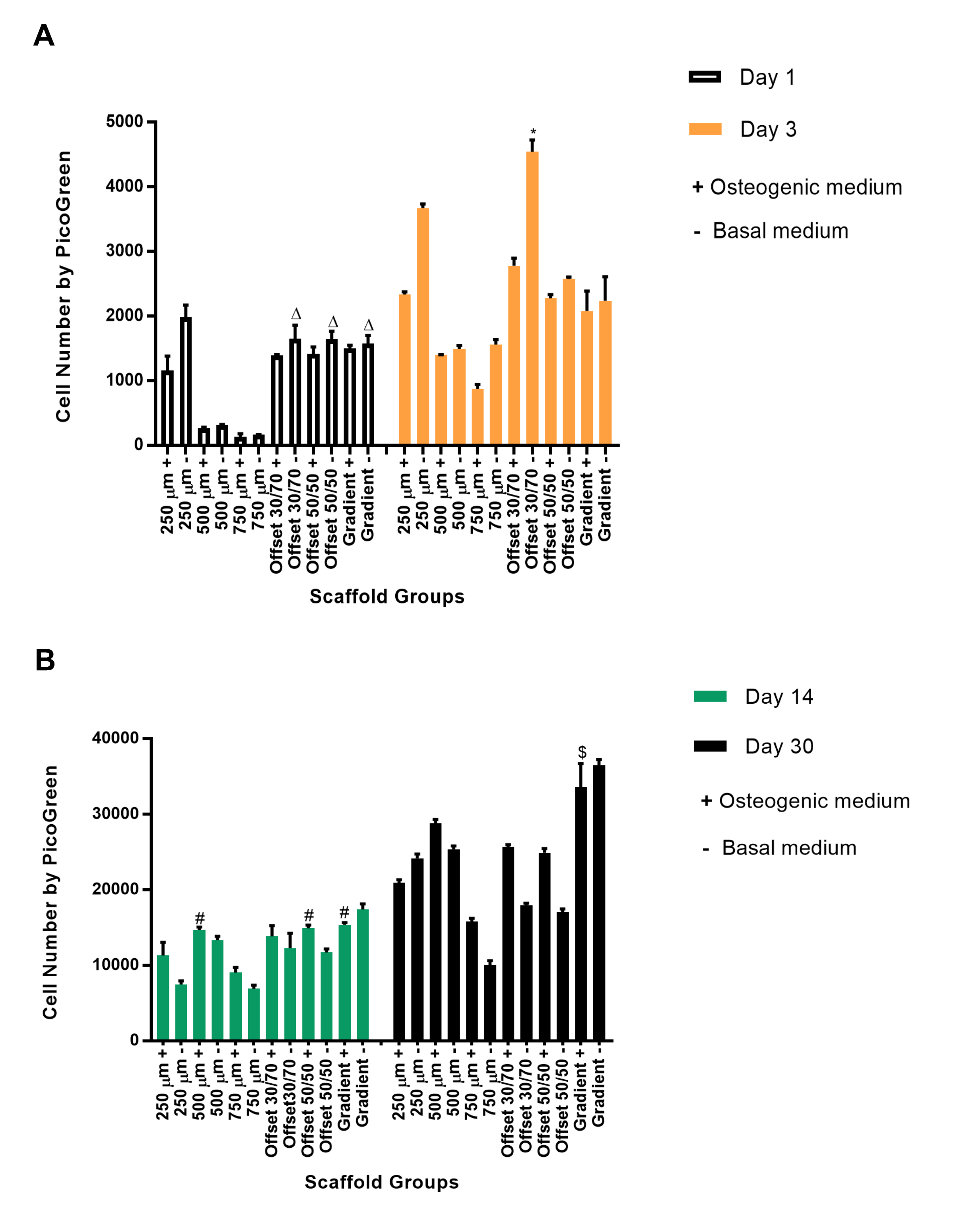


**Additional file 1.**
