# Supplementary material for: Role of offset and gradient architectures of 3-D melt electrowritten scaffold on differentiation and mineralization of osteoblasts
Source: Biomater Res. 2020 Jan 3;24:2. doi: 10.1186/s40824-019-0180-z (PMC6942301; doi:10.1186/s40824-019-0180-z)
Supplement: Supplementary file 1 — Additional file 1. Osteoblast Proliferation. Proliferation of osteoblasts in osteogenic (+) and basal medium (−) seeded on PCL scaffolds with different porosity for 1, 3, 14, and 30 days. * significant versus other scaffolds. Δ nonsignificant versus 250 μm -. # and $ nonsignificant versus Gradient - (p < 0.01); (Reproduced with permission from Abbasi et.al. doi: https://doi.org/10.1021/acsbiomaterials.8b01456). [file 40824_2019_180_MOESM1_ESM.zip › Copyright for Additional file 1.pdf]

Effects of Gradient and Offset Architectures on the Mechanical and Biological Properties of 3-D Melt Electrowritten (MEW) Scaffolds

ACS Publications
Most Trusted. Most Cited. Most Read.

Author: Naghmeh Abbasi, Abdalla Abdal-hay, Stephen Hamlet, et al
Publication: ACS Biomaterials Science & Engineering
Publisher: American Chemical Society
Date: Jul 1, 2019

Copyright © 2019, American Chemical Society

PERMISSION/LICENSE IS GRANTED FOR YOUR ORDER AT NO CHARGE

This type of permission/license, instead of the standard Terms & Conditions, is sent to you because no fee is being charged for your order. Please note the following:

- Permission is granted for your request in both print and electronic formats, and translations.
- If figures and/or tables were requested, they may be adapted or used in part.
- Please print this page for your records and send a copy of it to your publisher/graduate school.
- Appropriate credit for the requested material should be given as follows: "Reprinted (adapted) with permission from (COMPLETE REFERENCE CITATION). Copyright (YEAR) American Chemical Society." Insert appropriate information in place of the capitalized words.
- One-time permission is granted only for the use specified in your request. No additional uses are granted (such as derivative works or other editions). For any other uses, please submit a new request.

If credit is given to another source for the material you requested, permission must be obtained from that source.

BACK CLOSE WINDOW
