# Supplementary figures and images for: Role of offset and gradient architectures of 3-D melt electrowritten scaffold on differentiation and mineralization of osteoblasts
Source: Biomater Res. 2020 Jan 3;24:2. doi: 10.1186/s40824-019-0180-z (PMC6942301; doi:10.1186/s40824-019-0180-z)

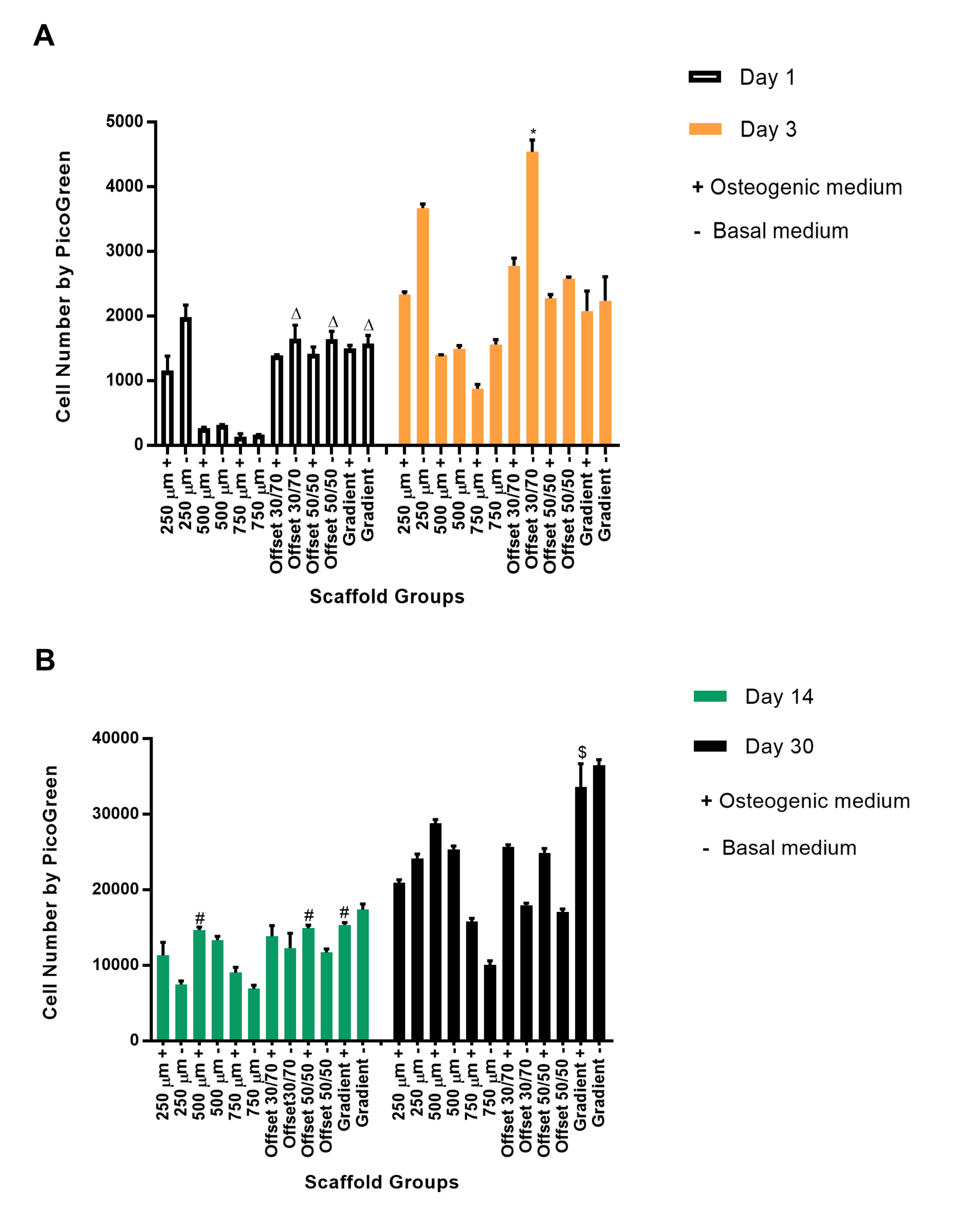

Supplement: Supplementary file 1 — Additional file 1. Osteoblast Proliferation. Proliferation of osteoblasts in osteogenic (+) and basal medium (−) seeded on PCL scaffolds with different porosity for 1, 3, 14, and 30 days. * significant versus other scaffolds. Δ nonsignificant versus 250 μm -. # and $ nonsignificant versus Gradient - (p < 0.01); (Reproduced with permission from Abbasi et.al. doi: https://doi.org/10.1021/acsbiomaterials.8b01456). [file 40824_2019_180_MOESM1_ESM.zip › Additional file 1.tif]
